# Dyslexia and Fonts: Is a Specific Font Useful?

**DOI:** 10.3390/brainsci8050089

**Published:** 2018-05-14

**Authors:** Christina Bachmann, Lauro Mengheri

**Affiliations:** 1 Studio Centro Risorse, 59100 Prato, Italy; 2 Studio Verbavoglio, 57100 Livorno, Italy; lauromengheri@verbavoglio.org

**Keywords:** dyslexia, EasyReading™, compensating tools, font

## Abstract

Nowadays, several books published in different fonts advertised as being particularly suitable for dyslexics are available on the market. Our research aimed to assess the significance of a specific reading font especially designed for dyslexia, called EasyReading™. The performances of good readers and dyslexics were compared. Fourth grade primary school students (533 students in total) were assessed based on reading tasks presented with two different layouts: the popular Times New Roman and EasyReading™, in order to investigate whether children’s performances were influenced by the fonts used. The results of the study were both statistically and clinically significant, proving that EasyReading™ can be considered a compensating tool for readers with dyslexia, and a simplifying font for all categories of readers.

## 1. Introduction

Specific learning disabilities (SLD), such as dyslexia, require help to facilitate scholastic pathways, but their handling varies from country to country; in many countries, having a dyslexia diagnosis means the attendance of special classes, with a different program and differentiated training objectives. In Italy, however, the diagnosis brings about the right to use certain instruments and measures (compensatory instruments and exemptions) without modifying the scholastic pathway, which proceeds within the same class as “normal” students, and without the support of specialized teachers, leading to the achievement of a normal diploma.

Thanks to Italian Law 170/2010, which contains new norms on learning disabilities [1], and to the guidelines on educational rights for students with specific learning disabilities (decree of Italian Ministry of Education D.M. 5669 of 12 July 2011) [2], the adoption of compensatory tools and dispensation measures has become an undeniable right for all students with specific learning disabilities (SLD) in Italy.

Since then, educational institutions have been required to integrate compensatory tools and dispensary measures into their education programs, and teachers have had to familiarize themselves with terms such as speech synthesis, digital spellcheckers, and personalized teaching plans. Having to implement special strategies in class, it becomes necessary for teachers to be able to transform the planned tasks for the class, and also to make them accessible to students with dyslexia.

Furthermore, school books had to become available not only in a hardcopy version, but also in a downloadable one (Circular of Ministry of Education C.M. 18 of 9 February 2012) [3], in order for the font to be accessible in the most suitable size for the reader, and above all, to be readable out loud by speech synthesis software. Even though information technology is widely recognized as an important reading aid, it cannot address all of the problems that dyslexics face every time they have to deal with a written text [4].

In addition to the use of vocal readers (synthesized voice that reads digital books) to transform a reading task into a listening task, dyslexic students also often find themselves facing some tasks in paper format, in which they are obliged to read. If these tasks could have graphic characteristics that facilitate reading, they could also become compensatory instruments for closing the gap between the deficient reading abilities of the dyslexic student and the rest of the class.

Several publishers are specializing in suitable fonts for dyslexics and are publishing books specially designed for different age ranges of this audience. Although it is important that the text outlet is simple and suitable for dyslexics, the content must be age-appropriate in order not to put the readers off or make them feel inept. Having books that are interesting, age-tailored and that only differ in their font can make reading enjoyable for people with reading difficulties. For these reasons, we examined a font that has been widely used in scholastic texts in Italy to verify whether it presents facilitating characteristics for readers with dyslexia, and whether it could, therefore, be useful to use in class test texts.

Developmental dyslexia (DD) is a neurodevelopmental disorder identified in about 5% of the student population. It impedes reading fluently and accurately. Children with DD read more slowly and inaccurately, although they have average intelligence, adequate access to conventional instruction, and an absence of neurological and/or psychiatric disorders.

There are different theories regarding the etiology of dyslexia, some of which are still hotly debated: the phonological awareness theory [5,6,7,8,9,10,11], the rapid auditory processing theory [12,13,14,15,16,17,18], the magnocellular-dorsal theory [19,20,21], and the attentional deficit theory [22,23,24,25,26,27,28].

In Italy, research on the effect of using different fonts is sparse. We know from international research that for subjects with average vision there is a small difference when changing the font from Times New Roman to Courier, but that this difference becomes significant in readers with weak vision [29]. Dyslexics do not have severely impaired vision, their low performance in reading is due to defects in language processing, like poor phonological awareness, or deficits in their visual system, e.g., crowding.. Visual crowding is an increased difficulty in correctly identifying stimuli—in the case of text, this relates to single letters—with reduced distance between the nearby letters. It mainly affects the peripheral vision of adults, while in children it mainly affects central vision. Obviously, knowing how to correctly recognize letters is one of the abilities necessary to learn to read at an early phase, and to read quickly and correctly in successive phases. Stronger crowding leads to the inability to recognize letters when other letters surround them, with the consequence of reading more slowly and making more mistakes [30].

Reading fluency is the main predictor of legibility, which depends on different typographic factors: the size of the character, the presence of serifs, the space between the lines, the space between the words, and the space between the letters. Bernard et al. found that Times New Roman and Arial are read faster than Courier, Schoolbook and Georgia, and that fonts at the 12-point size are read faster than fonts at the 10-point size [31]. Many studies show that serif fonts are more legible, and help to distinguish letter and words better [32], but other research shows that there is no difference between the legibility of serif and sans serif fonts [33]. The reduced space between letters due to the ornaments of serif fonts can lead to the crowding effect affecting reading fluency and accuracy [34,35]. 

Various studies have shown that subjects with developmental dyslexia suffer more from the effects of crowding. In opaque orthographies, phonological deficits are prominent, while Italians are native speakers in a transparent orthography. Martelli et al. [36] tested the hypothesis that crowding effects are responsible for the reading slowness characteristic of developmental dyslexia and found that abnormal crowding accounts for 60% of Italian dyslexics’ slow reading. 

While this study lacked a comparison with normal readers, one subsequent study verified that due to crowding, a simple manipulation of letter spacing can improve text reading performance in Italian and French individuals [37], which is congruent with a previous study by Spinelli et al. [38]. The authors examined the reading performance of 74 children between the ages of 8 and 14—namely, from the 3rd to the 9th grade—of which 34 were Italian and 40 were French, divided in two subgroups based on the order of administration of the two texts, the normal one, and another text with different spacing. They compared the performances with a control group of 30 normal reading children. The limited number of subjects participating in the research, the wide range of ages and the diversity of reference writing systems (Italian children, in fact, have a transparent writing system, while French children have a relatively opaque writing system) make it difficult to generalize these results, which seem to indicate that normal readers do not show any improvement in reading with an increase of letter spacing, in contrast to dyslexic readers. Therefore, there has been a lack of research that involves a representative number of subjects with homogeneous ages and clinical characteristics in comparing the performance between dyslexic students and normal readers.

Ruffino et al. [39] found that dyslexics with poor phonological decoding have both spatial and temporal attention difficulties, confirming the role of attention. In addition, they verified, in accordance with the phonological theory, that impaired phonological awareness correlates with impairment in reading of non-words [10,40,41,42,43], due to visual attention difficulties [39].

The publisher Angolo Manzoni created a specific font called EasyReading™ (Torino, Italy), which, thanks to its high graphical legibility, is able to satisfy the special needs of dyslexic readers. EasyReading™ has a big size, a simple design, and a special serif, in order to help dyslexic people distinguish between letters and numbers of similar shapes (d-b, p-q, 6-9). Letter and word spacing, as well as line spacing and the spacing between words and punctuation marks, are wide. The text has no hyphenated words; it is not justified, and the line’s interruption follows a natural reading flow. All these auxiliary aids can be rightfully considered compensatory tools if they genuinely help to address the reading deficit and facilitate a more accurate and fluent performance. Italian and French publishers already use EasyReading™ in many textbooks (for example, Flammarion, De Agostini Scuola, Ed. Centro Studi Erickson, Pearson Italia). It is possible for everyone to easily install it on any computer (Microsoft, Apple) or tablet (iOS, Android) as an additional font in word processing software (for further information, please visit www.easyreading.it/en (English version)). According to the EasyReading™ creators, this font is suitable for people with LD because “it has specific graphic features that make reading easier for dyslexic people”. This statement, which is drawn from the Turin branch of the Italian Dyslexics Association (AID), has until now not been scientifically supported. 

In our clinical practice, we noticed that texts edited with the EasyReading™ font were extremely successful in helping children with dyslexia, as well as children with reading problems not related to SLD (specific learning disorder). Could reading really become easier by changing the font? The aim of this study is to answer this question by comparing reading performances obtained with the popular Times New Roman and EasyReading™ fonts. 

## 2. Materials and Methods

### 2.1. Participants

Sixteen primary schools, belonging to seven educational institutions in the Prato province (Italy), participated in the study. A total of 664 fourth-grade primary school students (364 males and 300 females) were recruited, of which 107 were foreign students. The final sample was of 533 children, because some were excluded: 12 children did not have their parents’ consent; a class of 20 children dropped out while the study was ongoing; 33 foreign children had been living in Italy for less than two years, and had too poor a knowledge of the language; 57 children were absent on the test days; and some children could not participate because of impairments (Italian Disability Law 104/92) [44].

The sample group who took part in the tests was composed of 533 fourth-grade students, 282 were males and 251 were females. The average age was 9.5 years (average expressed in months: 115 ± 4). The ethnicities of the children were: 456 children were Italian and 21 were Chinese (out of the 73 foreign students), which was the most sizeable foreign community in the research project area.

### 2.2. Tools

Children were tested on reading and non-verbal intelligence. We administrated three reading tasks: the excerpt for the 4th class from the MT reading test [45], and a word task and a non-word task derived from the DDE-2 battery [46]. To assess non-verbal intelligence and exclude intellectual developmental disorders, we used the Raven’s Colored Progressive Matrices CPM [47]. 

**Figure 1:**
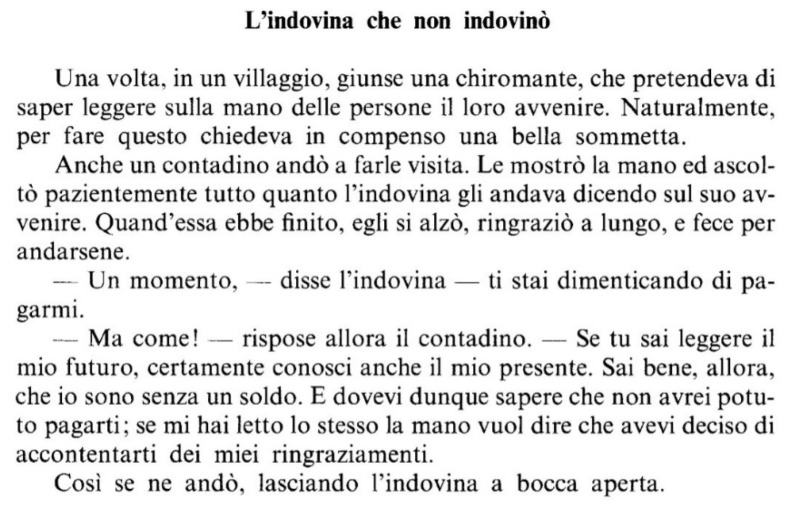
Times New Roman version.

**Figure 2:**
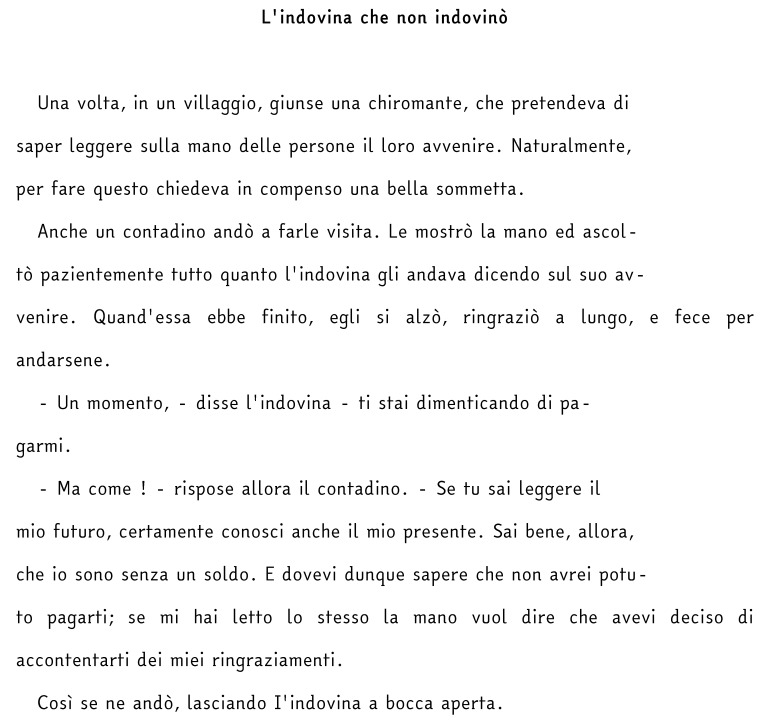
EasyReading™ version.

All reading tests (text, lists of words and non-words) were used in their original version (MT and DDE-2) and in a modified version specially prepared for this study, in which the original Times New Roman font was replaced with the EasyReading™ font. In order not to create further elements of diversity, the number of syllables per line, the graphic layout and the character size were kept the same among all tests. The only aspects that differed were those specific to the EasyReading™ font, such as line spacing, letter spacing and the lack of serifs (Figure 1 and Figure 2).

### 2.3. Procedure

Each child took part in three sessions; the reading tests were undertaken during the first and second sessions, and the Raven CPM matrices during the third. 

The reading tests were given in two different orders, while the Raven CPM matrices were always administered at the end: 

1st order: excerpt in the original font, word and non-word reading tasks in the original font, excerpt in the EasyReading™ font, word and non-word reading tasks in the EasyReading™ font, CPM; 

2nd order: excerpt in the EasyReading™ font, word and non-word reading tasks in the EasyReading™ font, excerpt in the original font, word and non-word reading tasks in the original font, CPM.

All tests were undertaken individually and were administered by psychologists. 

For the MT excerpt reading test, we referred to the new norms of Cornoldi et al. [48], to the latest manual edition for the word and non-word reading tasks derived from the DDE-2 test [46], and to the Italian normative data manual [49] for the Raven CPM matrices.

### 2.4. Sample Group Description

The sample was divided into four groups according to the points scored on the original versions of the MT and DDE-2 reading tests, as follows: 

Group 0 (normal readers): scores above the 25th percentile at the CPM and average scores in the reading test; 

Group 1 (reading difficulties): scores above the 25th percentile at the CPM and reading skills performances below average (fluency between 1 and 2 standard deviation below average and/or accuracy between 15th and 5th percentile);

Group 2 (dyslexia: students already diagnosed with dyslexia or pinpointed as dyslexic during the testing): scores above the 25th percentile in the CPM and two or more deficit performances in the reading test (fluency more than 2 standard deviation below average and/or accuracy below the 5th percentile);

Group 3 (CPM below average): scores below or equal to the 25th percentile in the CPM test.

According to these criteria, 426 children had no reading problems (group 0, normal readers), 27 children had some difficulties in reading (group 1), 54 children were dyslexic (group 2), and 26 children required further investigation regarding their intellectual functioning (group 3) (Table 1).

## 3. Results

Average and standard deviation scores were collected for the overall sample and for each single group. The order effect was not considered in the final scoring, as it was not statistically significant.

According to the MT test ranges, four different categories emerged: fully achieved criteria (over the 75th percentile), sufficient performance (between the 16th and 74th percentile), below average (between the 6th and 15th percentile), and clinical range (under the 5th percentile).

Students with difficulties in reading were 1.3% when the reading text was presented in the original font; this dropped to 0.2% when it was submitted in the EasyReading™ version. In fact, 20 children scored below average in reading fluency performance when the text was presented in Times New Roman; 13 below average (within 2 standard deviations) and seven within the clinical range (more than 2 standard deviations below average). Only eight scored below average performance when the text was in the EasyReading™ font (seven within 2 standard deviations and one below 2 standard deviations) (Table 2).

The EasyReading™ font also had an important influence on reading accuracy; while 12 students were in the clinical range using the original text, this number decreased to nine when the EasyReading™ version was used.

Furthermore, of the 54 children with a diagnosis of dyslexia (10.1% of the total students), only 27 (5.1% of the total) still fulfilled the criteria for dyslexia when the assessment was made using the EasyReading™ font (Table 3).

Hereafter, reading fluency (syllables per second) and accuracy were compared in the performances obtained with the original Times New Roman version and with the EasyReading™ one. In the EasyReading™ version, the average fluency was 4.16 syllables per second with a standard deviation of 1.09, while in the Times New Roman version it was 3.50 syllables per second with a standard deviation of 0.94 (statistically significant difference; *t*_(531)_ = −32.12, *p* < 0.001). 

A similar significant difference was also found when comparing the performances in the word and non-word reading tasks; in the word task, the average reading fluency was 3.03 in the original version and went up to 3.33 (*t*_(532)_ = −18.14, *p* < 0.001) in the EasyReading™ one, while in the non-word task, it increased from 1.86 to 2.04 (*t*_(532)_ = −10.37, *p* < 0.001) (Table 4).

Accuracy significantly improved in the word and non-word tasks, but not in the reading excerpt. In the word task, students’ mistakes decreased from 5.49 on average (using the original format) to 4.14 (*t*_(532)_ = 9.56, *p* < 0.001) in the EasyReading™ format, while in the non-word task, mistakes were reduced from 7.72 to 6.49 (*t*_(532)_ = 8.41, *p* < 0.001) (Table 5).

Reading fluency significantly improved within all groups when the text was presented in the EasyReading™ version. 

Focusing on each group, it is possible to notice that normal readers had an average reading fluency of 3.73 syllables per second, falling within the fully achieved performance criteria range. Dyslexics read at an average fluency of 2.67 syllables per second, with a performance in the sufficient performance range. Children with reading difficulties read at an average fluency of 2.39 syllables per second and had a performance in the sufficient performance range, too. Finally, students with low non-verbal intelligence scored 2.63 syllables per second on average, which meant that they fell within the sufficient performance range.

In the EasyReading™ version, normal readers scored 4.44 in the reading fluency (syllables per second), an improvement of 0.71 syllables per second (*t*_(424)_ = −30.52, *p* < 0.001). Dyslexics read 3.19 syllables per second, gaining 0.52 syllables per second (*t*_(53)_ = −8.64, *p* < 0.001). Children with reading difficulties increased their fluency by 0.51 syllables per second (*t*_(26)_ = −6.82, *p* < 0.001) reading 2.90 syllables per second. Finally, students with low nonverbal intelligence gained 0.36 syllables per second (*t*_(25)_ = −4.77, *p* < 0.001), as they read 2.99 syllables per second (Table 6).

In the EasyReading™ version, reading accuracy significantly improved for the dyslexic group, in which errors were reduced from 6.59 to 6.25 (*t*_(53)_ = −3.43, *p* < 0.001). This trend was also observed in those with reading difficulties, whose mistakes decreased from 5.83 to 5.50 (*t*_(26)_ = 0.74, *p* < 0.001). Reading accuracy got worse in the other two groups (Table 7).

Concerning the word and non-word tasks (DDE-2 test), the discussion focused only on normal readers and dyslexics. Please refer to the tables for data related to other groups.

In the list of words, dyslexic children significantly improved their reading fluency in the EasyReading™ version compared to the original one, increasing from 2.19 syllables per second to 2.39 (*t*_(53)_ = −6.38, *p* < 0.001). The same applied to normal readers, who read the original version in 3.26 syllables per second and the EasyReading™ version in 3.58 (*t*_(425)_ = −16.37, *p* < 0.001) (Table 6).

Accuracy also improved in both groups when using EasyReading™; in fact, reading mistakes were reduced from 13.35 in the original version for dyslexics to 9.93 in the EasyReading™ one (*t*_(53)_ = 4.94, *p* < 0.001), and for normal readers it dropped from 3.69 to 2.78 (*t*_(425)_ = 7.22, *p* < 0.001) (Table 7).

A similar trend was also found in the list of non-words, where reading fluency, as well as accuracy, improved for both groups in the EasyReading™ version.

Dyslexic children read 1.42 syllables per second (Times New Roman font) and at 1.58 syllables per second (EasyReading™ font), showing an improvement of 0.16 syllables per second (*t*_(53)_ = −4.85, *p* < 0.001), while normal readers read the first font at 1.96 syllables per second and the second at 2.16, therefore showing an improvement of 0.20 s in the case of the EasyReading™ version (*t*_(425)_ = −13.16, *p* < 0.001) (Table 6).

Furthermore, in term of accuracy, reading mistakes decreased from 14.22 to 10.61 for dyslexic readers (*t*_(53)_ = 15.30, *p* < 0.001) and from 6.31 to 5.50 for normal readers (*t*_(425)_ = 5.74, *p* < 0.001) (Table 7).

## 4. Discussion 

The purpose of this research was to check whether reading becomes easier for dyslexics when changing the font from Times New Roman to EasyReading™. The results show a statistically relevant difference between the performances; the EasyReading™ font resulted in a positive impact on reading fluency across all reading tests (excerpt, words, and non-words). EasyReading™ was particularly useful for dyslexic children, who also scored significantly better in reading accuracy. The EasyReading™ format helped students improve their reading performances without requiring any training (phonological, attentional or orthographic). The results confirm the hypothesis, but in future research, it will be necessary to clarify whether they depend on the specific font, the size of the font, or the spacing between the letters, the words and the lines. 

We eliminated the training effect (test-retest effect) by means of the different presentation order used in the experimental design; the data show that there were no statistically significant differences. Regarding font size, the characters of the stimuli were of the same size. We can therefore reasonably conclude that much of the effect could be due to the spacing, even though the weight of the specific characteristics of the EasyReading^TM^ font is still not clear and will require additional work.

Recent research on a font named “Dyslexie”, which is used in many primary schools in the Netherlands, concluded that the increase in size and space facilitates reading in children with reading difficulties, regardless of the font used [50]. Given that in this research, they compared this specific font with the Arial font, such results cannot be generalized to a font with different characteristics, such as EasyReading™. In fact, the comparison of EasyReading™ with another font (Times New Roman) has instead shown that it was able to increase the performance of children with dyslexia, both in fluency and accuracy, for all stimuli presented (excerpt, words and non-words); in dyslexic children, it increases reading fluency and leads to fewer errors. Normal readers’ performance improves too, in contrast to the outcomes of previous research [37].

It is not clear whether a Times New Roman version with expanded spacing could produce the same results. The reading fluency of dyslexic children benefits from increased spacing and font size [35,37], because dyslexics are more vulnerable to visual crowding [30,36,38]. Consistent with the research already mentioned in the introduction [33,34,35], the presence of serifs in Times New Roman could reduce the space between letters and words, so we can assume that it does not receive the same improvements in terms of legibility as a sans serif font. Future research should investigate the difference between EasyReading™ and spaced Times New Roman to answer this question. In fact, a limitation of this research is that a third version of the stimuli using Times New Roman with expanded spacing is missing. 

The reading fluency improvement (syllables per second) resulting from the EasyReading™ font is statistically and clinically significant. In fact, reading fluency improvements of 0.16 for the non-words and of 0.52 for the excerpt are larger than the natural annual improvement. Longitudinal studies show that in a year, a dyslexic person makes an improvement of 0.30 syllables per second for excerpts and 0.14 for non-words [51,52], which are smaller than the improvements resulting from EasyReading™ alone.

## 5. Conclusions

These results are important for several reasons. 

Firstly, students read more easily with EasyReading™, as demonstrated by the improvement in their reading fluency and accuracy. For this reason, reading tasks should be given in this format, rather than in Times New Roman.

Secondly, teachers can facilitate reading for normal and dyslexic readers by simply changing fonts when preparing exams and texts for their students. The clinical improvement resulting from EasyReading™ is so consistent as to overtake the natural reading improvement in a year, thus proving that EasyReading™ makes reading easier. This provides the opportunity for dyslexic students to partially fill the gap between their reading fluency and that of their classmates, just by using this font. 

Thirdly the dyslexic sample was 10.1% of the total students in the current study, which is double that of data reported by epidemiological studies. In this regard, it is important to highlight that the assessment made in order to pinpoint the students with specific reading disabilities cannot be considered to be as precise or adequate as that used to make a proper dyslexia diagnosis. In fact, the latter requires a more complex and accurate clinical assessment, considering not only inclusion criteria, like in the present study, but also exclusion criteria, as recommended by the Consensus Conference of the Italian National Institute of Health (ISS, Istituto Superiore di Sanità) [53] and the ICD-10 of the World Health Organization [54].

In this research, it was not difficult to assess IQ with a multi-component test or to investigate exclusion factors. Nevertheless, by excluding students with fewer than two years of schooling and all children with a poor knowledge of Italian, those with a lack of education were excluded. By excluding differently abled children, the possibility of the tests being influenced by cognitive or sensorial impairments was decreased. In addition, setting the cut-off of the Raven Matrix above the 25th percentile helped to rule out children with underdiagnosed cognitive deficits. Unfortunately, it was not possible to exclude the influence of emotional, social or cultural problems which could have affected the children’s performances. Regardless of all the above considerations, it remains important to investigate the reasons why so many students failed the reading tests, as it is unlikely for them all to be dyslexic. 

Finally as the reading fluency and accuracy improvements were appreciable across all groups (normal readers, readers with difficulties, dyslexics and students with cognitive difficulties), EasyReading™ should be considered an important aid for all students.

The decoding process, in which many processes and abilities are involved, is complex, such that there is no agreement within the scientific community on the cause of dyslexia. In dyslexic students, we only observe the symptom, namely inadequate reading, but clinicians know from experience that a single function is not deficient. It is necessary to further study all the processes that may explain low reading performance, seeking to integrate various theories that examine different aspects of the same phenomenon. It remains to be seen which of the individual characteristics makes EasyReading^TM^ an ideal font for both dyslexics and normal readers, without excluding the hypothesis that it may actually be the simultaneous presence of more elements (line spacing, letter spacing and the lack of serifs) that determines the improvement effect on reading ability.

In conclusion, based on the evidence collected and the consistent considerations, even though the weight of the specific characteristics of the EasyReading^TM^ font is still not clear and will require additional work, the EasyReading™ font facilitates reading for normal and dyslexic readers. Therefore, it can rightfully be considered a very effective compensating tool for dyslexia and a facilitating font for all readers.

## Figures and Tables

**Table 1:** Division of the sample into four groups according to the points scored on the original versions of the MT and DDE-2 reading tests: group 0—normal readers; group 1—children with some reading difficulties; group 2—children with dyslexia; and group 3—children with low non-verbal intelligence.

Group 0 (Normal Readers)	Group 1 (Reading Difficulties)	Group 2 (Dyslexia)	Group 3 (CPM below 25th Percentile)	Total
426	27	54	26	533

**Table 2:** Number of students included in the performance range in according to the MT manual.

Version	Fully Achieved Criteria		Sufficient Performance		Below Average		Clinical Range	
	Fluency	Accuracy	Fluency	Accuracy	Fluency	Accuracy	Fluency	Accuracy
Times New Roman	235	308	278	172	13	41	7	12
EasyReading™	363	271	162	208	7	45	1	9

**Table 3:** Students in the clinical range for dyslexia.

Version	Frequencies	Percentages
Times New Roman	54	10.1%
EasyReading™	27	5.1%

**Table 4:** Reading fluency (syllables per second) in the reading tests.

Reading Task	Times New Roman	EasyReading™
Excerpt (*t*_(531)_ = −32.12, *p* < 0.001)	3.50 ± 0.94	4.16 ± 1.09
Words (*t*_(532)_ = −18.14, *p* < 0.001)	3.03 ± 0.88	3.33 ± 0.93
Non-words (*t*_(532)_ = −10.37, *p* < 0.001)	1.86 ± 0.60	2.04 ± 0.61

**Table 5:** Reading accuracy (errors) in the reading tests.

Reading Task	Times New Roman	EasyReading™
Excerpt (*t*_(532)_ = −2.62, *p* < 0.001)	3.10 ± 2.75	3.34 ± 2.90
Words (*t*_(532)_ = 9.56, *p* < 0.001)	5.49 ± 5.32	4.14 ± 4.55
Non-words (*t*_(532)_ = 8.41, *p* < 0.001)	7.72 ± 5.30	6.49 ± 4.67

**Table 6:** Mean (M) and standard deviations (SD) of fluency (syllables per second) in reading tasks (excerpt, words and non-words) in Times New Roman (TNR) and EasyReading™ (ER) among all groups: Group 0 (normal readers), Group 1 (reading difficulties), Group 2 (dyslexic), Group 3 (low non-verbal intelligence).

Group	Excerpt		Comparison		Word		Comparison		Non-Word		Comparison	
	TNR M ± SD	ER M ± SD	*t*	*p*	TNR M ± SD	ER M ± SD	*t*	*p*	TNR M ± SD	ER M ± SD	*t*	*p*
0	3.73 ± 0.90	4.44 ± 0.92	*t*_(424)_ = −3.52	<0.001	3.26 ± 0.74	3.58 ± 0.79	*t*_(425)_ = -16.37	<0.001	1.96 ± 0.50	2.16 ± 0.56	*t*_(425)_ = −13.16	<0.001
1	2.39 ± 0.54	2.90 ± 0.75	*t*_(26)_ = -6.82	<0.001	1.98 ± 0.50	2.27 ± 0.60	*t*_(26)_ = −6.68	<0.001	1.55 ± 1.24	1.48 ± 0.44	*t*_(26)_ = 1.94	<0.001
2	2.67 ± 0.92	3.19 ± 1.13	*t*_(53)_ = −8.64	<0.001	2.19 ± 0.81	2.39 ± 0.83	*t*_(53)_ = -6.38	<0.001	1.42 ± 0.49	1.58 ± 0.53	*t*_(53)_ = -4.85	<0.001
3	2.63 ± 1.08	2.99 ± 1.14	(*t*_(25)_ = −4.77	<0.001	2.11 ± 0.92	2.26 ± 0.83	*t*_(25)_ = -2.65	<0.001	1.43 ± 0.59	1.53 ± 0.60	*t*_(25)_ = -2.39	<0.001

**Table 7:** Mean (M) and standard deviations (SD) of accuracy (errors) in reading tasks (excerpt, words and non-words) in Times New Roman (TNR) and EasyReading™ (ER) among all groups: Group 0 (normal readers), Group 1 (reading difficulties), Group 2 (dyslexic), Group 3 (low non-verbal intelligence).

Group	Excerpt		Comparison		Word		Comparison		Non-Word		Comparison	
	TNR M ± SD	ER M ± SD	*t*	*p*	TNR M ± SD	ER M ± SD	*t*	*p*	TNR M ± SD	ER M ± SD	*t*	*p*
0	2.27 ± 0.80	2.58 ± 1.96	*t*_(425)_ = −3.44	<0.001	3.69 ± 3.25	2.78 ± 3.01	*t*_(425)_ = 7.22	<0.001	6.31 ± 4.19	5.50 ±3.94	*t*_(425)_ = 5.74	<0.001
1	5.83 ± 2.57	5.50 ± 2,57	*t*_(26)_ = 0.74	<0.001	9.26 ± 4.25	7.22 ± 5.03	*t*_(26)_ = 2.36	<0.001	10.26 ± 3.91	8.67 ± 4.64	*t*_(26)_ = 1.94	<0.001
2	6.59 ± 3.97	6.25 ± 3.61	*t*_(53)_ = 0.90	<0.001	13.35 ± 5.40	9.93 ± 5.67	*t*_(53)_ = 4.94	<0.001	14.22 ± 5.71	10.61 ± 5.39	*t*_(53)_ = 5.64	<0.001
3	6.60 ± 4.06	7.50 ± 5.27	*t*_(25)_ = −1.33	<0.001	14.88 ± 6.77	11.19 ± 5.18	*t*_(25)_ = 4.33	<0.001	14.73 ± 5.51	11.85 ± 5.27	*t*_(25)_ = 3.69	<0.001
